# Beyond Folate: The Emerging Role of Maternal Vitamin B12 in Neural Tube Development

**DOI:** 10.3390/nu17122040

**Published:** 2025-06-19

**Authors:** Lirong Nie, Xinru Liu, Xiaoxue Li, Ziyang Ren, Xiao Cheng, Yuwei Wu, Zhiwen Li, Jufen Liu

**Affiliations:** 1 Institute of Reproductive and Child Health, National Health Commission Key Laboratory of Reproductive Health, Peking University, No 38 College Rd, Haidian District, Beijing 100191, China; nielirong@bjmu.edu.cn (L.N.); ziyang_ren@bjmu.edu.cn (Z.R.); stchengxiao@hotmail.com (X.C.); wuyuwei@stu.pku.edu.cn (Y.W.); lizw@bjmu.edu.cn (Z.L.); 2 Department of Epidemiology and Biostatistics, School of Public Health, Peking University, No 38 College Rd, Haidian District, Beijing 100191, China; 3 School of Public Health, Peking University, No 38 College Rd, Haidian District, Beijing 100191, China; liuxinru@stu.pku.edu.cn; 4 Department of Health Education, Cangzhou City Center for Disease Prevention and Control, Cangzhou 061000, China

**Keywords:** vitamin B12, folic acid, neural tube defects, meta-analysis

## Abstract

**Background/Objectives:** Folic acid (FA) supplementation can effectively reduce the occurrence of neural tube defects (NTDs). Vitamin B12 is involved in folate metabolism; however, studies have not reached a definitive conclusion on the association between vitamin B12 and NTDs independent of folate levels. A systematic review and meta-analysis were performed to summarize existing research and investigate the effect of vitamin B12 on NTDs. **Methods:** Studies were systematically searched in PubMed, Web of Science, Embase, and Cochrane, published before 1 March 2024. Standardized mean difference (SMD) with 95% confidence interval (CI) was employed to assess the association between maternal vitamin B12 in blood and NTDs. **Results:** A total of 38 studies were included, with a total of 2316 NTDs and 4298 controls, covering 14 countries worldwide. Compared with the non-NTD group, the NTD group exhibited a lower vitamin B12 level [SMD = −0.23, 95% CI (−0.32, −0.14), *p* < 0.001, *I*^2^ = 58.3%] with a statistically significant difference. Additionally, there was a significant association between maternal vitamin B12 concentration and NTDs when there was no significant difference in folate between the NTD and control groups [SMD: −0.19, 95% CI (−0.28, −0.10)]. **Conclusions:** Vitamin B12 supplement is also essential for the prevention of NTDs besides folic acid. Monitoring vitamin B12 concentration among pregnant women and considering appropriate supplementation with a combination of vitamin B12 and folic acid could be explored.

## 1. Introduction

Neural tube defects (NTDs), common types of birth defects, have received considerable attention in the past few decades and have been greatly prevented [1]. However, in 2021, the global incidence of NTDs was still up to 93.47 per 100,000 live births [2], and low sociodemographic index regions (e.g., Eastern Sub-Saharan Africa) were the most affected [3]. Therefore, NTDs remain a serious public health concern worldwide given their substantial role in perinatal mortality.

It is well established that folic acid (FA) supplementation during the periconceptional period can prevent NTDs [1], as countries implementing FA supplementation or mandated FA fortification have reported a marked decrease in NTD prevalence [4,5,6]. Vitamin B12 is involved in folate metabolism, and its deficiency can impair the conversion and utilization of methylated folate [7,8]. The biochemical speciation of vitamin B12 in the human body is complex, the assessment of which employs multiple biomarkers. Total B12, holo-transcobalamin (holo-TC), methylmalonic acid (MMA), and homocysteine, as sensitive metabolic indicators, could be used to screen for vitamin B12 status [9].

Biologically, vitamin B12 can encompass DNA synthesis and regulation, neurological maintenance, and erythropoiesis [10], all of which are particularly critical during pregnancy, necessitating elevated dietary intake of vitamin B12 to counteract the physiological decline in serum B12 concentrations [11]. Emerging evidence has suggested that high intakes of one-carbon cofactors, such as vitamin B12, were associated with reducing NTD risk in the offspring among mothers meeting folic acid recommendations [12,13]. A study also found that vegetarian dietary habits were associated with suboptimal B12 intake and resulted in a 1.6-fold increased NTD risk [14]. Furthermore, among pregnant women, concentrations of MMA were higher in women who gave birth to children with NTDs than in the control group (130 vs. 105 nM), with a strong dose-dependent association with NTD risk [15]. Holo-TC II levels were related to the risks of having a child with an NTD [16], and pregnant women who gave birth to children with NTDs had significantly lower serum or plasma vitamin B12 levels [17,18,19,20,21]. However, certain studies reported an association between maternal vitamin B12 and NTD risk without adequately controlling for folate’s influence, making it impossible to determine vitamin B12’s specific role [18,22,23]. In addition to contemporary shifts in dietary patterns (e.g., increased prevalence of vegetarianism) and lifestyle behaviors (e.g., weight management practices), further investigation into the role of vitamin B12 in NTD etiology remains essential.

To address these research gaps, we performed this systematic review to explore the association between maternal vitamin B12 status and NTDs.

## 2. Materials and Methods

### 2.1. Search Strategy

This systematic review and meta-analysis strictly adhered to the Preferred Reporting Items of Systematic Reviews and Meta-Analyses (PRISMA) guidelines recommendations. We comprehensively searched PubMed, Web of Science, Embase, and Cochrane to identify all relevant studies published before 1 March 2024. Databases were searched by following terms: vitamin B12; b 12 vitamin; cyanocobalamin; cobalamins; cobalamin; methylcobalamin; eritron; neural tube defects; NTDs; developmental neural tube defects; anencephaly; brain congenital absence; anencephalus; incomplete anencephaly; spina bifida; spinal dysraphism; encephalocele; meningocele; myelomeningocele; lipomyelomeningocele; hydranencephaly. The total search strategy is listed in Table S1.

### 2.2. Study Selection and Data Extraction

A study was included when it was (1) a cohort study or case–control study, or (2) a study that showed a comparison of serum vitamin B12 or plasma vitamin B12 concentrations in women with NTD-affected pregnancy and women without NTD-affected pregnancy.

Studies were excluded when (1) their title, abstract, or full text was not relevant to the topic under study; or (2) they were reviews, conference abstracts, letters, patents, meta-analyses, or academic thesis; or (3) their full text was not available; or (4) they were cross-sectional study, intervention experiments or animal experiments; or (5) they were not in English; or (6) they had no relevant values for concentrations of vitamin B12, folate, or other study variables.

**Figure 1:**
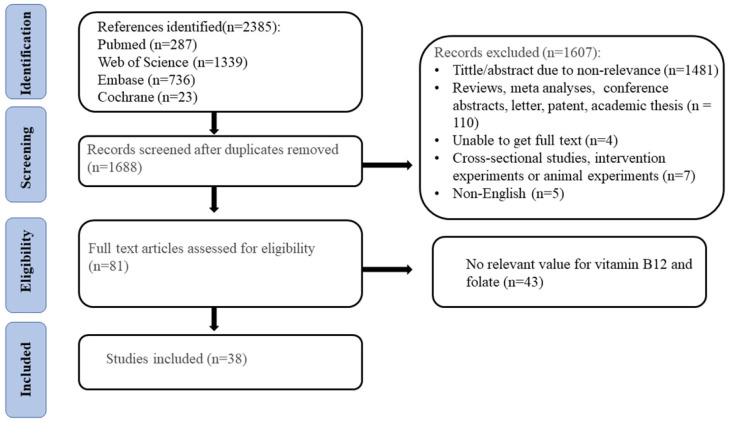
Flow diagram of the study search and selection process.

Two reviewers (LN and XL [Xinru Liu]) independently assessed publications for eligibility. Eventually, this study involved 43 studies that met the inclusion criteria (Figure 1). From all eligible articles, two reviewers independently extracted data from each study based on a pre-designed Excel table. Data included the name of the first author, year of publication, nationality of participants, study design, number of participants (NTD/non-NTD), time of specimen collection, folate between the NTD and control groups (significant/non-significant), and the method for the determination of B12 levels. Quality was assessed by using the Newcastle–Ottawa Scale. If any discrepancies were found, a third reviewer would join the review.

Given the established critical role of folate in NTDs, we accounted for its effect by performing stratified analyses. One subgroup was defined by non-significant differences in maternal folate between the NTD and control groups, and another subgroup was defined by significant differences in maternal folate between the NTD and control groups. The analysis of the subgroup without significant differences in folate revealed the effect of vitamin B12 on NTD risk.

### 2.3. Statistical Analysis

This study was conducted using R software (version 4.3.3). All the results are demonstrated as standardized mean differences (SMDs) with 95% confidence intervals (CIs) for continuous variables (concentrations of vitamin B12) between the NTD group and the control group. Sensitivity analysis was conducted to assess the robustness of the synthesized results. The *p* value < 0.05 was considered statistically significant in all tests. The 11 studies provided only the median and interquartile range, so the approximate mean and standard deviation were calculated [24]. Given the assessment of vitamin B12 samples over different periods, it was expected that there would be considerable heterogeneity in all results. The *I*^2^ statistic was used to evaluate the heterogeneity among studies. *I*^2^ values >50% indicated substantial heterogeneity, and therefore subgroup analyses were further conducted to explore the cause. Publication bias was identified by the symmetry of the visualized funnel plot and assessed by Egger’s linear regression method.

## 3. Results

Following systematic screening, a total of 2385 studies were initially identified. Ultimately, 38 studies met the inclusion criteria, comprising 2316 cases of NTD-affected pregnancies and 4298 controls across 14 countries. The characteristics of the included studies are illustrated in Table 1. All the included studies were case–control studies. Among the 38 studies, 26 found no significant differences in maternal folate [19,20,21,25,26,27,28,29,30,31,32,33,34,35,36,37,38,39,40,41,42,43,44,45,46,47], whereas 12 revealed significant differences in maternal folate [18,22,23,48,49,50,51,52,53,54,55,56]. Overall, the specimen collection time was inconsistent, including during the second or third trimester, throughout the entire pregnancy, and during the postpartum period. Additionally, some studies did not specify the timing of collection at all. Additionally, of the 38 studies, 3 were from Africa (including Egypt and Ethiopia), 7 were from America (including Mexico, US–Mexico border, US, and southern Brazil), 9 were from Asia (including China, northern Iran, and India), and 19 were from Europe (including Turkey, Ireland, France, The Netherlands, UK, Finland, and Scotland) (Figure S1).

**Figure 2:**
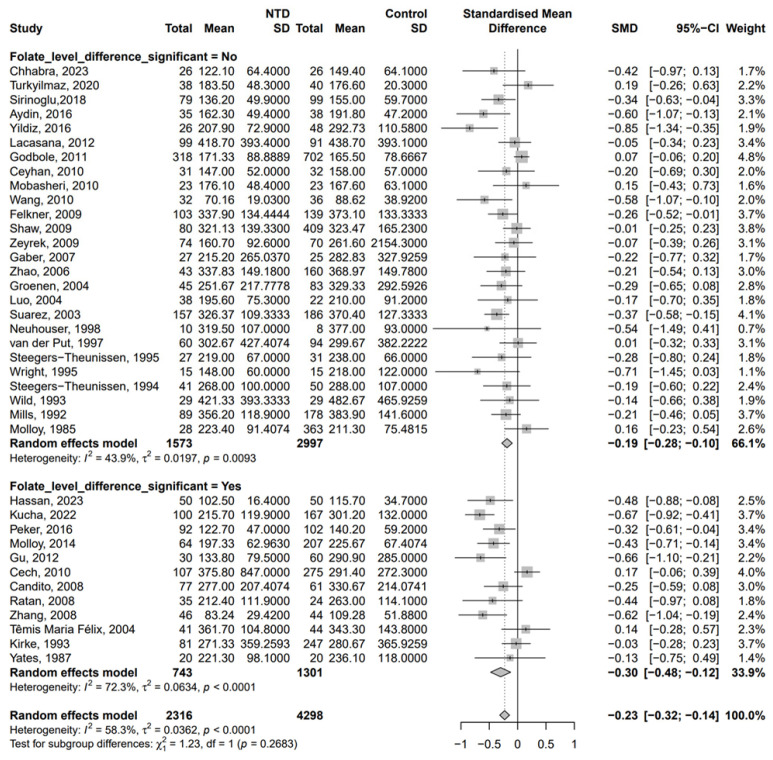
SMDs of vitamin B12 concentration (pmol/L) in the studies on NTDs [18,19,20,21,22,23,25,26,27,28,29,30,31,32,33,34,35,36,37,38,39,40,41,42,43,44,45,46,47,48,49,50,51,52,53,54,55,56].

The SMDs of maternal vitamin B12 concentration (pmol/L) between the NTD and control groups are demonstrated in Figure 2. Compared with the non-NTD group, a significantly lower concentration of vitamin B12 was found in the NTD group [SMD = −0.23, 95% CI (−0.32, −0.14), *p* < 0.001, *I*^2^ = 58.3%]. Due to the presence of moderate heterogeneity, a random-effect model was applied. There was publication bias in the analysis (Figure S2), and the result of Egger’s test was significant (*p* = 0.013).

**Figure 3:**
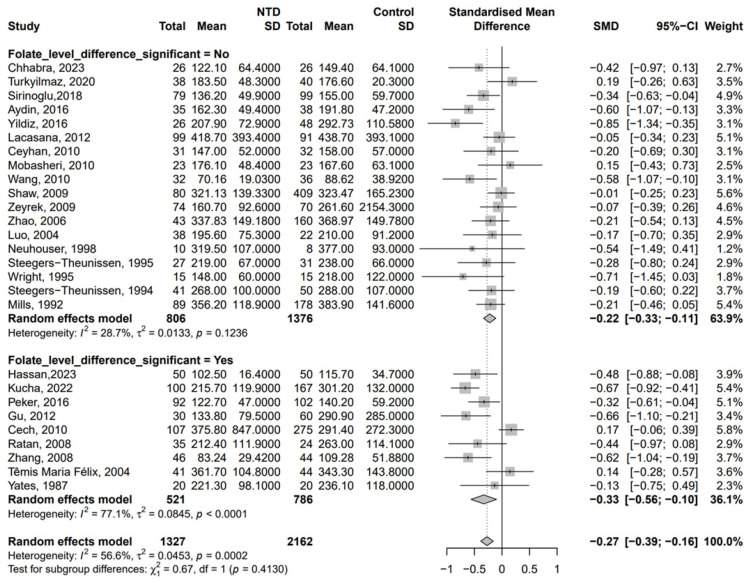
Sensitivity analysis: SMD of vitamin B12 concentration (pmol/L) on NTDs after excluding studies that provided only the median and interquartile range [18,19,20,21,22,23,25,26,27,28,30,31,32,33,36,37,40,42,43,44,46,49,50,51,52,54,56].

In the sensitivity analysis, we excluded studies that provided only the median and interquartile range (Figure 3). The results remained consistent, with lower B12 concentrations in the NTD group [SMD = −0.27, 95% CI (−0.39, −0.16), *p* < 0.001, *I*^2^ = 56.6%]. There was no publication bias in the sensitivity analysis (Figure S3), and Egger’s test was non-significant (*p* = 0.107), indicating that the asymmetry of the original funnel plot was more likely to be caused by data conversion errors than by selective publication. This supports the reliability of our findings despite variability in data reporting formats.

**Figure 4:**
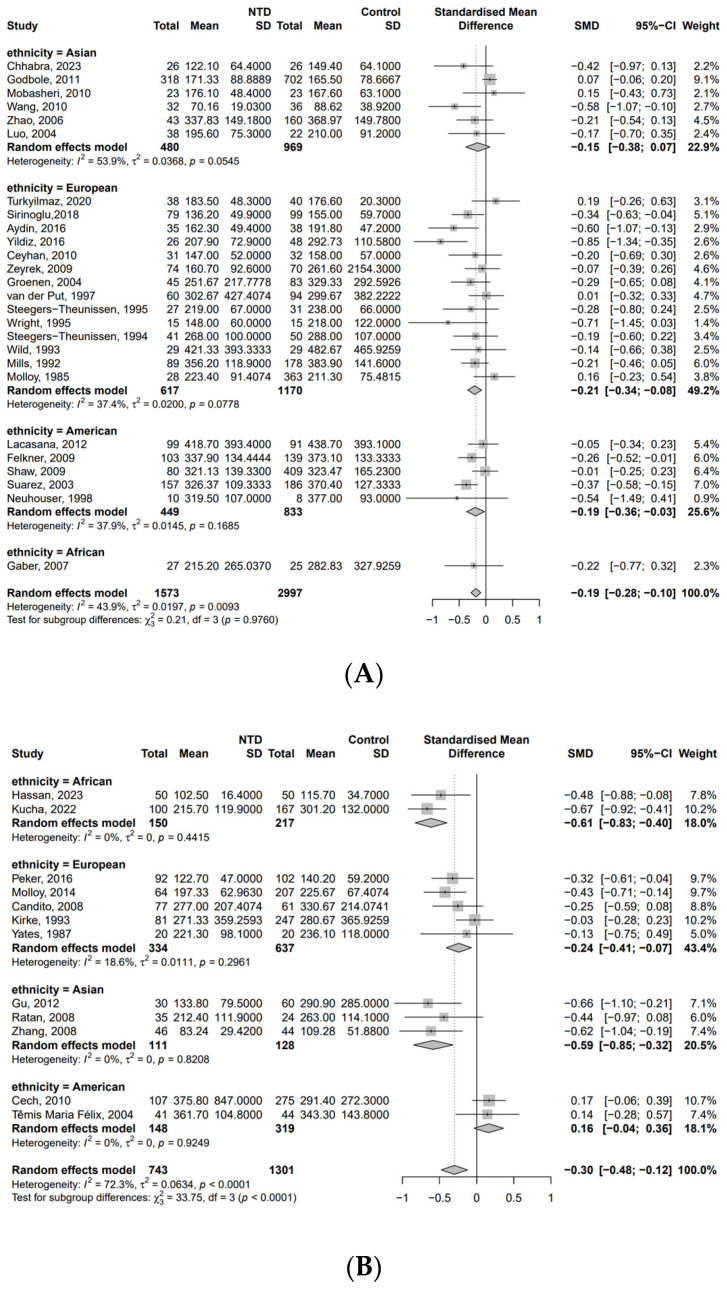
SMDs of vitamin B12 concentration (pmol/L) between the NTD and control groups: (**A**) among subjects with no significant differences in maternal folate between the NTD and control groups [19,20,21,25,26,27,28,29,30,31,32,33,34,35,36,37,38,39,40,41,42,43,44,45,46,47]; (**B**) among subjects with significant differences in maternal folate between the NTD and control groups [18,22,23,48,49,50,51,52,53,54,55,56].

In the subgroup analysis (Figure 2), there was a significant association between the maternal vitamin B12 concentration and NTDs when there was no significant difference in folate between the NTD and control groups [SMD: −0.19, 95% CI (−0.28, −0.10)]. A similar trend was found when there was a significant difference in folate between the NTD and control groups [SMD = −0.30, 95% CI (−0.48, −0.12)]. We further analyzed ethnicity (Figure 4). When there was no significant difference in folate, the concentration of vitamin B12 was lower in the NTD group among Europeans [SMD = −0.21, 95% CI (−0.34, −0.08)] and Americans [SMD = −0.19, 95% CI (−0.36, −0.03)] (Figure 4A). Meanwhile, the group with significant differences in folate showed lower vitamin B12 concentration in the NTD group among Africans [SMD = −0.61, 95% CI (−0.83, −0.40)], European [SMD = −0.24, 95% CI (−0.41, −0.07)], and Asians [SMD = −0.59, 95% CI (−0.85, −0.32)] (Figure 4B). Furthermore, different methods for the determination of B12 levels also had a certain effect (Figure S4).

## 4. Discussion

In this systematic review and meta-analysis, we revealed a statistically significant reduction in vitamin B12 concentration among mothers with NTD-affected pregnancy compared to controls, which was consistent with previous meta-analytic findings [57]. We further controlled for maternal folate, and when there was no significant difference in folate between the NTD and control groups, vitamin B12 was lower in the NTD group, suggesting an independent role of vitamin B12.

Vitamin B12 and folate are closely interrelated micronutrients that play essential roles in one-carbon metabolism [58]. The predominant circulating form of folate, 5-methyltetrahydrofolate (5-MTHF), serves as a cofactor in the remethylation of homocysteine to methionine, a reaction catalyzed by methionine synthase using vitamin B12 as a cofactor [59]. The resulting methionine is essential for the synthesis of key biomolecules, including creatine, phospholipids, proteins, neurotransmitters, and methylated nucleic acids [59]. Insufficient B12 levels may impair folate metabolism. The methyl trap hypothesis could best explain the interrelationship between these two vitamins, which holds that vitamin B12 deficiency impairs methionine synthase activity, thereby blocking the conversion of 5-methyltetrahydrofolate to biologically active tetrahydrofolate required for DNA biosynthesis and cell division [60,61]. Thus, cells suffer from a kind of folate pseudo-deficiency, which means adequate folate stores are rendered functionally unavailable for critical methylation processes and nucleotide biosynthesis, predisposing to NTD pathogenesis [60]. Additionally, high folic acid intake can resolve the anemia associated with vitamin B12 deficiency, potentially delaying the detection or treatment of the underlying B12 deficiency and even resulting in negative health outcomes [62]. Due to the close connection between folate and vitamin B12, special attention should be paid to supplementation. Furthermore, genetic factors also play a crucial role in the relationship between NTDs, folate, and vitamin B12. Deshmukh’s study identified a strong association between the MTHFR C677T polymorphism and NTDs [63]. Additionally, polymorphisms in the genes involved in vitamin B12 absorption and metabolism are associated with NTDs, such as mutations in exons 1 and 3 of the gastric intrinsic factor gene, variants in the lipoprotein-related protein 2 gene, alterations in the transcobalamin receptor gene, and the well-documented C677T polymorphism in the methylenetetrahydrofolate reductase (MTHFR) gene, which is in part related to vitamin B12 deficiency [28,64,65,66,67]. However, given the inconsistent data on the effect of vitamin B12 on NTDs, future clinical trials using folic acid and vitamin B12 supplements are necessary.

**Figure 5:**
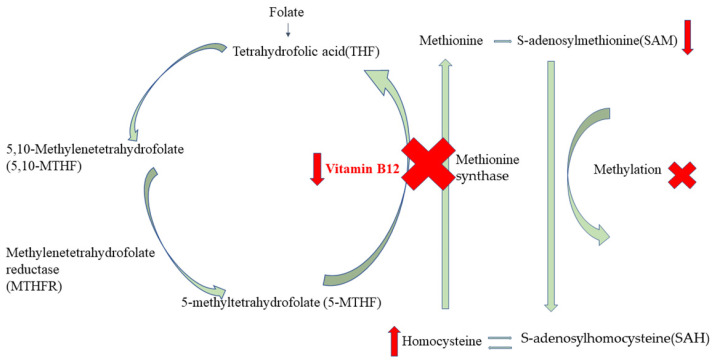
Associations between the folate cycle, vitamin B12, and methylation.

It is worth noting that vitamin B12 showed a downward trend in the NTD group independent of folate levels in our study. Vitamin B12, acting as a cofactor for methionine synthase and homocysteine remethylation, regulates two biochemically consequential pathways (Figure 5). Firstly, insufficient vitamin B12 could compromise the synthesis of S-adenosylmethionine (SAM), a universal methyl donor required for methylation reactions involving DNA, RNA, histones, proteins, neurotransmitter metabolism, and membrane phospholipid synthesis [68]. Aberrant methylation patterns from SAM depletion could disrupt neural tube closure mechanisms [69]. Secondly, low vitamin B12 could cause elevated homocysteine levels, which could exert direct embryotoxic effects and damage endothelial and smooth muscle cells independently of folate status [70,71]. Lower vitamin B12, alongside elevated homocysteine in pregnancies, has been identified as a potential biomarker of NTD risk [22]. High homocysteine in mothers with NTDs can impact DNA methylation and synthesis in the developing fetus. This disruption affects the genes that regulate the closure of the neural tube [72]. It is worth noting that further increases in homocysteine concentration are toxic, generating free radicals through auto-oxidation which pose additional risks to fetal development [73].

Overall, despite widespread prenatal supplementation, low vitamin B12 still exists. In low- and middle-income countries, including India, Bangladesh, South Africa, and Croatia, 26% to 51% of pregnant women had vitamin B12 deficiency [74]. Meanwhile, vitamin B12 deficiency is not confined to low-income countries [75,76]. Vitamin B12 is naturally found only in animal-derived foods. It might be because of poverty and limited access to foods containing vitamin B12, as well as cultural or religious customs, health pursuits, environmental problems, and personal restrictions (e.g., vegetarianism), that such foods are deliberately avoided [77,78,79]. Studies have shown significantly lower cobalamin levels in vegans compared to non-vegans, and a European study reported a high prevalence of inadequate dietary vitamin B12 intake among vegan populations [80,81]. Genetic factors have an influence on vitamin B12 status. Higher concentrations of total vitamin B12 were found in black compared with white populations [82]. Furthermore, in China, a population-based cross-sectional study involving 1170 women further illustrated that the total prevalence of vitamin B12 deficiency was 45.5%, which indicated that vitamin B12 deficiency remained prevalent [83]. Similarly, among pregnant women in eastern Turkey, 12.5% exhibited low folate concentrations, 33.7% exhibited vitamin B12 deficiency alone, and 29.8% had both deficiencies concurrently [17]. Consequently, attention to monitoring vitamin B12, especially in women of reproductive age, is of great importance.

To the best of our knowledge, this study is a comprehensive meta-analysis that covers a larger number of studies and a wider area across 14 countries, including developed countries and developing countries, delving into the association of vitamin B12 with NTDs. Secondly, studies were stratified based on whether there was a statistically significant difference in folate between the NTD group and the control group to assess the independent association between vitamin B12 levels and NTDs. Finally, we conducted a sensitivity analysis to further enrich and support our findings. The results revealed a similar direction and effect with the main analysis.

This study has limitations. Firstly, as the included studies were designed as case–control studies, further evidence based on a cohort study was needed. Secondly, the current study mainly focused on serum or plasma vitamin B12 status as the main biomarker. Although this is a commonly used and important indicator, other active forms of vitamin B12 in the human body were not taken into account. Future research should integrate other complementary indicators (e.g., methylmalonic acid, holo-transcobalamin) to comprehensively evaluate B12 status.

## 5. Conclusions

In conclusion, this systematic review and meta-analysis revealed that the NTD group exhibited a lower vitamin B12 level, especially when there was no significant difference in folate between the NTD and control groups, suggesting an independent role of vitamin B12. It may be beneficial to monitor vitamin B12 concentrations among pregnant women, and the consideration of appropriate supplementation with a combination of vitamin B12 and folic acid could be explored based on individual needs and clinical recommendations.

## Figures and Tables

**Table 1:** Characteristics of the included studies (*n* = 38).

First Author	Publication Year	Country	Number of Participants (NTD/Non-NTD)	Time of Specimen Collection ^a^	Folate Between NTD and Control ^b^	Method for Determination of B12
Chhabra	2023	India	26/26	Postpartum	0	ELISA
Hassan	2023	Egypt	50/50	Pre-pregnancy	1	ELISA
Kucha	2022	Ethiopia	100/167	Second or third trimester of pregnancy	1	ELISA
Turkyilmaz	2020	Turkey	38/40	Second trimester of pregnancy	0	Radioimmunoassay
Sirinoglu	2018	Istanbul/Turkey	79/99	Second trimester of pregnancy	0	Chemiluminescence immunoassay
Aydin	2016	Turkey	35/38	Second trimester of pregnancy	0	Chemiluminescence immunoassay
Yildiz	2016	Turkey	26/48	Pre-pregnancy	0	Chemiluminescence immunoassay
Peker	2016	Turkey	92/102	Postpartum	1	Competitive Immunoassay
Molloy	2014	Ireland	64/207	Second trimester of pregnancy	1	Microbiological assays
Gu	2012	China	30/60	Second or third trimester of pregnancy	1	ELISA
Lacasana	2012	Mexico	99/91	Postpartum	0	ELISA
Godbole	2011	India	318/702	All gestational weeks	0	Microbiological assays
Cech	2010	US–Mexico border	107/275	Not mentioned	1	Radioimmunoassay
Ceyhan	2010	Turkey	31/32	Second trimester of pregnancy	0	Radioimmunoassay
Mobasheri	2010	Northern Iran	23/23	Second trimester of pregnancy	0	Radioimmunoassay
Wang	2010	China	32/36	All gestational weeks	0	Competitive Immunoassay
Felkner	2009	US–Mexico border	103/139	Postpartum	0	Competitive Immunoassay
Shaw	2009	US	80/409	Second or third trimester of pregnancy	0	Mass spectrometry
Zeyrek	2009	Turkey	74/70	Second or third trimester of pregnancy	0	ELISA
Candito	2008	France	77/61	All gestational weeks	1	Radioimmunoassay
Ratan	2008	India	35/24	Postpartum	1	Chemiluminescence immunoassay
Zhang	2008	China	46/44	All gestational weeks	1	Chemiluminescence immunoassay
Gaber	2007	Egypt	27/25	Pre-pregnancy	0	Radioimmunoassay
Zhao	2006	China	43/160	Postpartum	0	Radioimmunoassay
Têmis Maria Félix	2004	southern Brazil	41/44	Pre-pregnancy	1	Radioimmunoassay
Groenen	2004	The Netherlands	45/83	Pre-pregnancy	0	Chemiluminescence immunoassay
Luo	2004	China	38/22	Second trimester of pregnancy	0	Chemiluminescence immunoassay
Suarez	2003	US–Mexico border	157/186	Postpartum	0	Competitive Immunoassay
Neuhouser	1998	US	10/8	Pre-pregnancy	0	Radioimmunoassay
Van der Put	1997	The Netherlands	60/94	Pre-pregnancy	0	Radioimmunoassay
Steegers-Theunissen	1995	The Netherlands	27/31	Second trimester of pregnancy	0	Radioimmunoassay
Wright	1995	Northern Ireland	15/15	Postpartum	0	Radioimmunoassay
Steegers-Theunissen	1994	The Netherlands	41/50	Postpartum	0	Radioimmunoassay
Kirke	1993	Ireland	81/247	All gestational weeks	1	Microbiological assays
Wild	1993	UK	29/29	Postpartum	0	Radioimmunoassay
Mills	1992	Finnish	89/178	All gestational weeks	0	Radioimmunoassay
Yates	1987	Scotland	20/20	Pre-pregnancy	1	Competitive Immunoassay
Molloy	1985	Dublin	28/363	All gestational weeks	0	Microbiological assays

^a^ Time of specimen collection: pre-pregnancy refers to those who have a history of NTD-affected pregnancy. ^b^ Folate comparison between the NTD and control groups: 0 indicates no significant difference in maternal folate between the NTD and control groups, and 1 indicates a significant difference in maternal folate between the NTD and control groups.

## Data Availability

Data is contained within the article or Supplementary Materials.

## Supplementary Materials

The following supporting information can be downloaded at: https://www.mdpi.com/article/10.3390/nu17122040/s1, Figure S1: Country/regional distribution of included studies; Figure S2: Funnel plots for effect of vitamin B12 concentration (pmol/L) on NTDs; Figure S3: Sensitivity analysis: Funnel plots for effect of vitamin B12 concentration (pmol/L) on NTDs after excluding studies that provided only the median and interquartile range; Figure S4: SMDs of vitamin B12 concentration (pmol/l) on NTDs by different methods for determination of B12; Table S1: Search strategy in the study.
