# Supplementary material for: Beyond Folate: The Emerging Role of Maternal Vitamin B12 in Neural Tube Development
Source: Nutrients. 2025 Jun 19;17(12):2040. doi: 10.3390/nu17122040 (PMC12196037; doi:10.3390/nu17122040)
Supplement: Supplementary file 1 [file nutrients-17-02040-s001.zip › nutrients-3587301-supplementary.pdf]

Supplementary materials

Table Legends

Table S1. Search strategy in the study 2

Figure S1. Country/regional distribution of included studies 4

Figure S2. Funnel plots for SMD of vitamin B12 concentration (pmol/l) on NTDs 5

Figure S3. Sensitivity analysis: Funnel plots for SMD of vitamin B12 concentration (pmol/l) on NTDs after excluding studies that provided only the median and interquartile range 6

Figure S4. SMDs of vitamin B12 concentration (pmol/l) on NTDs by different methods for determination of B12 7

Table S1. Search strategy in the study

**Database: Pubmed**
Searching terms:<br>1： (((((((((vitamin B 12[MeSH Terms])) OR (b 12 vitamin[Title/Abstract])) OR (vitamin b12[Title/Abstract]))OR(b12 vitamin[Title/Abstract])) OR (Cyanocobalamin[Title/Abstract])) OR (Cobalamins[Title/Abstract])) OR (Cobalamin[Title/Abstract])) OR (hydroxocobalamin[Title/Abstract])) OR (methylcobalamin[Title/Abstract])) OR (Eritron[Title/Abstract]) 34378<br>2: (((((((((((((((((((neural tube defects[MeSH Terms]) OR (NTDs[Title/Abstract])) OR (defect, neural tube[Title/Abstract])) OR (developmental neural tube defects[Title/Abstract])) OR (anencephaly[Title/Abstract])) OR (brain congenital absence[Title/Abstract])) OR (anencephalus[Title/Abstract])) OR (incomplete anencephaly[Title/Abstract])) OR (partial anencephaly[Title/Abstract])) OR (spina bifida[Title/Abstract])) OR (spinal dysraphism[Title/Abstract])) OR (open spine[Title/Abstract])) OR (cleft spine[Title/Abstract])) OR (rachischisis[Title/Abstract])) OR (encephalocele[Title/Abstract]))) OR (meningocele[Title/Abstract])) OR (myelomeningocele[Title/Abstract])) OR (lipomyelomeningocele[Title/Abstract])) OR (hydranencephaly[Title/Abstract]) 38516<br>#3: 1 and 2
**Database: Embase**
Searching terms:<br>1: 'vitamin b 12'/exp OR 'b 12 vitamin':ab,ti OR 'vitamin b12':ab,ti OR 'b12 vitamin':ab,ti OR cyanocobalamin:ab,ti OR cobalamins:ab,ti OR cobalamin:ab,ti OR hydroxocobalamin:ab,ti OR methylcobalamin:ab,ti OR eritron:ab,ti<br>2 : ('neural tube defects')/exp OR ((NTDs):ab,ti) OR (('defect, neural tube'):ab,ti) OR (('developmental neural tube defects'):ab,ti) OR ((anencephaly):ab,ti) OR (('brain congenital absence'):ab,ti) OR ((anencephalus):ab,ti) OR (('incomplete anencephaly'):ab,ti) OR (('partial anencephaly'):ab,ti) OR (('spina bifida'):ab,ti) OR (('spinal dysraphism'):ab,ti) OR (('open spine'):ab,ti) OR (('cleft spine'):ab,ti) OR ((rachischisis):ab,ti) OR ((encephalocele):ab,ti) OR ((meningocele):ab,ti) OR ((myelomeningocele):ab,ti) OR ((lipomyelomeningocele):ab,ti) OR ((hydranencephaly):ab,ti)<br>#3: 1 and 2
**Database: Web of science**
Searching terms:<br>1:((((((((((TS=(vitamin B 12)) OR AB=(b 12 vitamin)) OR AB=(vitamin b12)) OR AB=(b12 vitamin)) OR AB=(Cyanocobalamin)) OR AB=(Cobalamins)) OR AB=(Cobalamin)) OR AB=(hydroxocobalamin)) OR AB=(methylcobalamin)) OR AB=(Eritron)) NOT (SILOID==("PPRN"))<br>2: (((((((((((((((((((TS=(neural tube defects)) OR AB=(NTDs)) OR AB=(defect, neural tube)) OR AB=(developmental neural tube defects)) OR AB=(anencephaly)) OR AB=(brain congenital absence)) OR AB=(anencephalus)) OR AB=(incomplete anencephaly)) OR AB=(partial anencephaly)) OR AB=(spina bifida)) OR AB=(spinal dysraphism)) OR AB=(open spine)) OR

AB=(cleft spine)) OR AB=(rachischisis)) OR AB=(encephalocel)) OR AB=(meningocele)) OR AB=(myelomeningocele)) OR AB=(lipomyelomeningocele)) OR AB=(hydranencephaly)) NOT (SILOID==("PPRN"))<br>#3： 1 and 2
**Database: Cochrane Library**
Searching terms:<br>#1 ("vitamin B12"):ti,ab,kw OR (hydroxocobalamin):ti,ab,kw OR (methylcobalamin):ti,ab,kw OR ("cyanocobalamin"):ti,ab,kw OR ("cobalamins"):ti,ab,kw<br>#2 (neural tube defects):ti,ab,kw OR (anencephaly):ti,ab,kw OR (spina bifida):ti,ab,kw OR (encephalocele):ti,ab,kw<br>#3 #1 AND #2

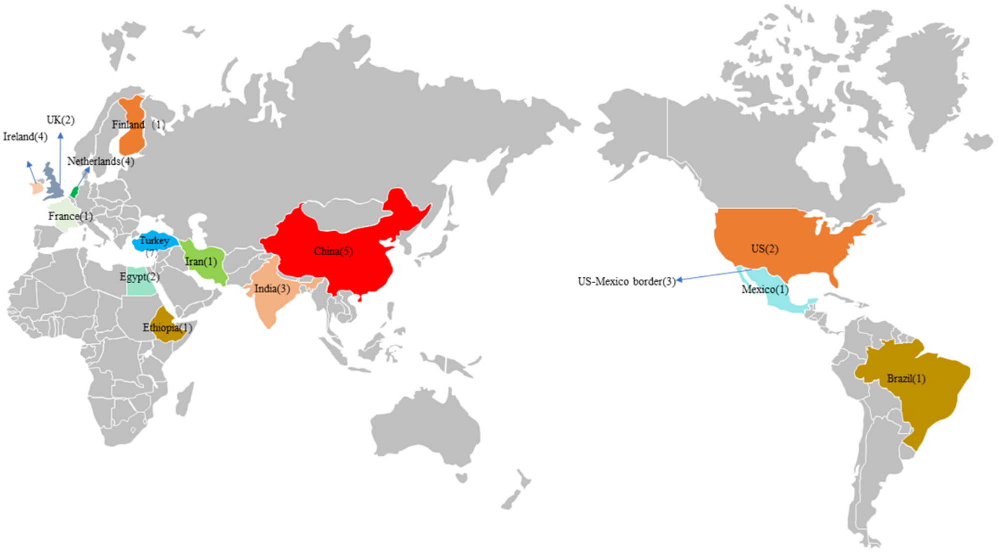

Figure S1. Country/regional distribution of included studies

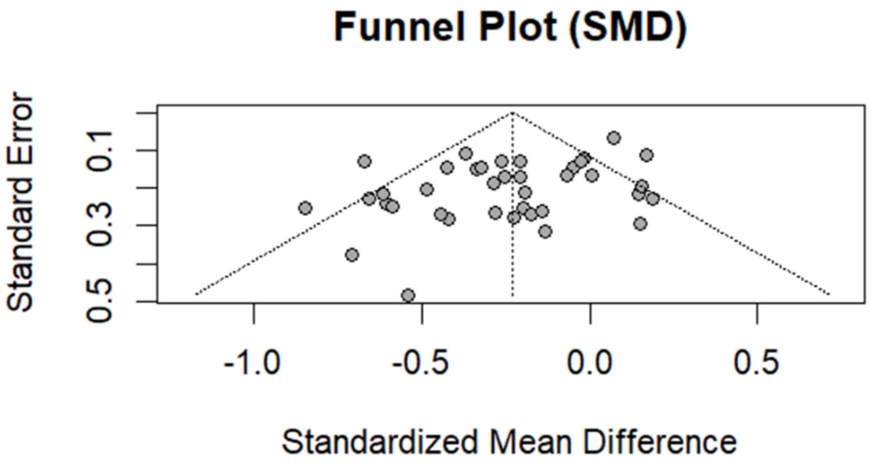

Figure S2. Funnel plots for SMD of vitamin B12 concentration (pmol/l) on NTDs

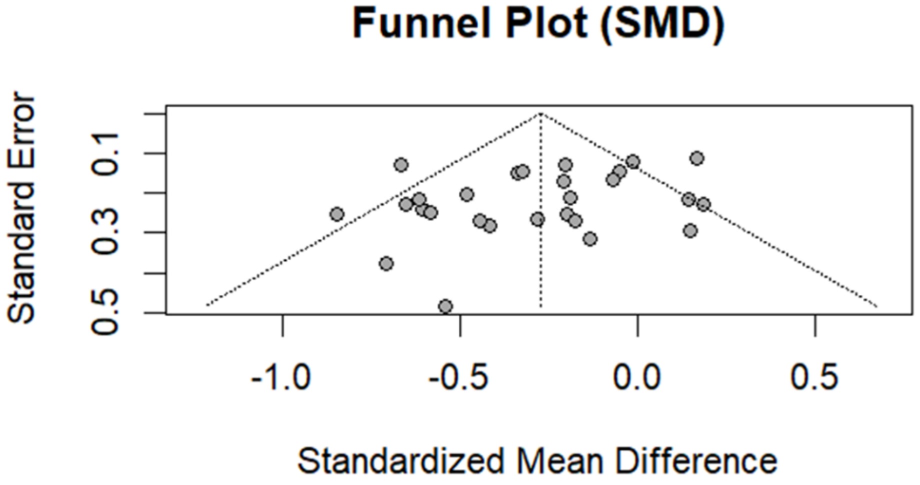

Figure S3. Sensitivity analysis: Funnel plots for SMD of vitamin B12 concentration (pmol/l) on NTDs after excluding studies that provided only the median and interquartile range

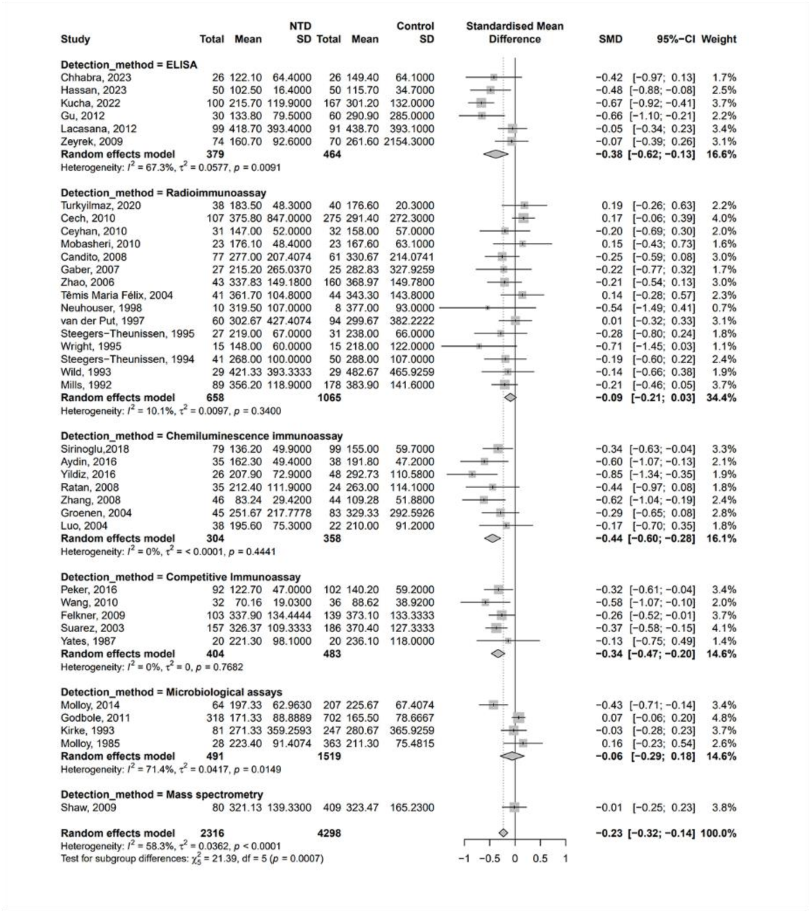

Figure S4. SMDs of vitamin B12 concentration (pmol/l) on NTDs by different methods for determination of B12
